# Oncologic Outcomes of Salvage Surgery in Recurrent Oral Tongue Squamous Cell Carcinoma

**DOI:** 10.7759/cureus.58403

**Published:** 2024-04-16

**Authors:** Soroush Farsi, Sharon Amole, Deanne King, Vural Emre, Jumin Sunde, Mauricio Moreno

**Affiliations:** 1 Department of Otolaryngology – Head and Neck Surgery, University of Arkansas for Medical Sciences, Little Rock, USA

**Keywords:** survival, outcomes, salvage, squamous cell carcinoma, salvage glossectomy, recurrent, oral tongue

## Abstract

Objective: This study aimed to determine the oncologic outcomes and identify prognostic factors in patients undergoing salvage glossectomy for recurrent oral tongue squamous cell carcinoma (OTSCC).

Methods: A retrospective chart review was conducted encompassing all patients who underwent salvage oral glossectomy out of 259 individuals undergoing oral glossectomy at a tertiary academic center. Inclusion criteria comprised patients who met the following conditions: 1) biopsy-proven oral tongue recurrence, 2) salvage glossectomy performed with curative intent, 3) availability of imaging records, and 4) comprehensive documentation. Cases involving base of tongue tumors and second primaries were excluded from the analysis. Categorical data were expressed as proportions, and continuous data as medians/quartiles. Univariate analysis used Fisher’s exact test for categorical variables and Student’s t-test for continuous ones. Survival analysis employed Kaplan-Meier estimates and the log-rank test.

Results: High-risk histopathological risk factors were significantly more common with recurrence compared to initial presentation. The mean locoregional disease-free interval was 35 months. Kaplan-Meier estimates for one- and three-year disease-free survival (DFS) were 62.7% and 33.4%, while disease-specific survival (DSS) rates were 73% and 38.9%, respectively. Recurrent T-stage was a predictor for DFS, while margin status was a strong predictor for both LR control (p = 0.024) and DSS (p = 0.030), as was perineural invasion (p = 0.001 and p = 0.030). Alcohol use was associated with worse overall survival (p = 0.024). In contrast to other reports, nodal status was not a predictor in this series.

Conclusions: Upon recurrence, histopathological analysis unveils detrimental changes in tumor biology, which significantly influence disease control. Notably, consistent with findings from other studies, factors, such as recurrent T-stage, presence of perineural invasion, and, most importantly, margin status, play pivotal roles in determining oncologic outcomes. Consequently, the imperative for aggressive salvage surgery becomes evident in achieving sufficient disease control. This underscores the necessity for proactive management strategies aimed at addressing these factors to enhance patient outcomes.

## Introduction

Oral cavity carcinomas rank as the 11th most prevalent malignancy globally, affecting various subsites within the oral cavity, with the oral tongue being the most frequently affected subsite [1]. Currently, oral tongue carcinomas compromise nearly 40% of all oral cavity cancers. Oral cavity squamous cell carcinoma (OCSCC) accounts for >90% of malignancies in the oral cavity, and the annual incidence of these cancers is estimated to be between four and 4.3 cases per 100,000 [2,3]. Unlike most other head and neck subsites, the incidence of oral tongue squamous cell carcinoma (OTSCC) has steadily increased over the last two decades [4]. The increase in OTSCC has predominantly been linked to Caucasian populations, particularly women. Potential causal factors may involve genetic abnormalities, such as Fanconi anemia, other oncogenic viral infections, and/or various environmental exposures [5].

Surgical resection with appropriately indicated adjuvant therapy remains the standard of care for oral cavity cancers. Despite appropriate therapy, the recurrence rate for oral cancers has been reported to range between 25% and 48% [6]. Patients with recurrent OTSCC are thought to be at imminent risk of succumbing to their disease. In addition, outcomes can vary drastically based on recurrent T-stage, presence of perineural invasion, margin status, and treatment response. Oftentimes, aggressive resection with adjuvant therapy is performed in order to obtain an oncologic cure [7]. Such interventions have major implications on patient’s speech, swallowing, and overall quality of life [8]. This places the surgeon and patient in a position to discuss prognosis and expectations from treatment options balanced against known risks of treatment.

In contrast to sites where organ-preservation approaches have become standard of care, there is a lack of data concerning the oncologic outcomes of salvage surgery in what remains a predominantly surgical disease. At present, interventions for recurrent OTSCC are provided with scant understanding of prognostic factors, mostly inferred from primary disease. Furthermore, another significant knowledge gap in the field of OTSCC concerns the tumor microenvironment and its pivotal influence on adverse histopathological features. The objectives of the study are clear: to identify prognostic factors for patients undergoing reoperative glossectomy for recurrent OTSCC, aiming to improve treatment strategies and patient outcomes. In addition, the study aims to contribute to future research by highlighting areas for the development of novel therapeutic approaches, particularly for high-risk recurrent OTSCC patients. Moreover, it seeks to understand the role of the tumor microenvironment in OTSCC recurrence and treatment resistance. Lastly, the study aims to assess if adverse histopathological features at initial presentation impact oncologic outcomes of salvage surgery, potentially refining risk assessment and personalized treatment planning. These objectives provide a comprehensive framework for the study's aims and potential contributions to the field.

This article was previously presented at the American Academy of Otolaryngology- Head and Neck Surgery Annual Meeting, San Diego, on September 18-21, 2016.

## Materials and methods

Subjects

This study was conducted at the University of Arkansas for Medical Science, a tertiary academic center in Little Rock, Arkansas, USA. The study design was approved by the university's Institutional Review Board (IRB# 228942). All study participants received salvage surgical therapy for recurrent OTSCC at the University of Arkansas for Medical within a period of 16 years. Inclusion criteria were defined as follows: 1) biopsy-proven recurrent OTSCC, 2) salvage glossectomy (all types) with curative intent, 3) axial imaging available for review, and 4) complete documentation of their therapy course. Exclusion criteria were defined as follows: 1) lesions involving the base of the tongue, 2) second primary tumors, 3) isolated nodal recurrences, and 4) imaging or complete documentation unavailable for review. Patients were not excluded based on the primary treatment received, and those treated with organ-preservation approaches were also included.

Data collection

A retrospective chart review was performed on all patients undergoing oral glossectomy throughout the defined study period. Subjects were selected according to the above-mentioned inclusion and exclusion criteria to isolate those patients receiving surgical salvage of recurrent OTSCC. All tumors were staged according to the American Joint Committee on Cancer TNM 2010 staging system [9] based on clinical assessment, review of available axial imaging, and pathology reports. All patients initially treated at an outside facility had complete pathological and axial imaging reports available for review. The chart review of all patient interactions from initial treatment to recurrence was reviewed to determine the time to recurrence. Data were collected from the medical records until oncologic surveillance was completed (five years post-treatment) or the time of death.

In order to identify patient-specific variables that are predictive of oncologic outcomes, 79 patient-specific variables were extracted from the chart review, including both patient- and tumor-specific variables from the time of both initial and recurrent treatments. These included patient demographics, tumor staging (initial and recurrent), type of initial treatment, adjuvant therapy received, initial histopathological findings, disease-free interval, type and extent of salvage glossectomy, preoperative histopathological findings, reconstruction technique, and oncologic outcomes.

Statistical analysis

The data analysis was generated using IBM SPSS Statistics for Windows, version 22.0 (released 2013, IBM Corp., Armonk, NY). The primary endpoints were recurrence-free survival and disease-specific survival. Categorical data were summarized as proportions, while continuous data were summarized as medians and quartiles. Univariate analysis was performed to establish the association between variables using Fisher's exact test for categorical variables, while Student's t-test was used for comparing means of continuous variables between two groups. Survival analysis was performed with Kaplan-Meier estimates and a log-rank test. A recurrence event was defined as clinical or radiological evidence of disease, with censoring at the last follow-up if no recurrence event occurred by then. For all statistical purposes, significance was defined as p < 0.05.

## Results

Patient demographics and treatment

The study cohort comprised 17 patients, eight (47.1%) males and nine (52.9%) females, with a mean age of 57.8 years (42-78, SD = 10.7); subject-specific demographics and tumor staging are presented in Table 1. The vast majority of patients (n = 15, 88%) were treated with primary surgery - most commonly a partial glossectomy (n = 12). Two subjects were initially treated with an organ-preservation approach consisting of chemoradiotherapy. Primary adjuvant radiotherapy (XRT) had been used in 46% of the cases (Table 2). Upon recurrence, a total or subtotal glossectomy was performed in eight (47.1%) cases; overall, reconstruction with free tissue transfer was necessary in nine (52.9%) patients. Adjuvant radiotherapy and chemotherapy were used in 65% and 47%, respectively following the salvage attempt. Among patients treated with adjuvant radiotherapy, 10 of 11 (91%) patients completed the full course (60 gray) with a single patient having to discontinue treatment secondary to severe mucocutaneous toxicity. It is worth mentioning the small sample size reported in this study and its potential effect on identifying and reporting significant findings, as well as on the power of the study.

**Table 1:** Summary of log-rank tests for initial and recurrent cohort variables impact on disease-specific survival. Statistically significant variables are presented in bold. P < 0.05 is considered significant.

Initial cohort variable	P-value	Recurrent cohort variable	P-value
Sex	0.457	Clinical T-stage	0.847
Smoking status	0.772	Glossectomy type	0.583
Alcohol use	0.229	Glossectomy extension	0.583
Clinical T-stage	0.014	Clinical N-stage	0.279
Clinical N-stage	0.005	Clinically positive N-status	0.486
Radiation therapy	0.486	Path T4-stage	0.010
Partial glossectomy	0.438	Path N-stage	0.152
Chemoradiotherapy	0.453	Tumor differentiation	0.187
Path T-stage	0.005	Well-differentiated	0.858
Path N-stage	0.688	Positive margin	0.029
Positive margins	0.338	Perineural invasion	0.001
Perineural invasion	0.087	Lymphovascular Invasion	0.893
Lymphovascular invasion	0.087	Extra-capsular spread	0.600
Advanced stage	0.486	Adjuvant radiotherapy	0.858
Free flap reconstruction	0.819	Local recurrence	0.232

**Table 2:** Intervention at the time of initial and salvage treatment. ND: neck dissection

Intervention	Number	Percentage
Initial treatment		
Surgery	10	59%
Organ preservation	2	12%
Surgery and radiation	5	29%
Salvage treatment		
Glossectomy	3	18%
Glossectomy + ND	3	18%
Glossectomy + adjuvant treatment	2	12%
Glossectomy, ND + adjuvant treatment	9	53%

Tumor staging and histological findings

At initial presentation, most patients had T1 and T2 lesions (n = 8 and n = 5, respectively), with 57% of patients being stage I or II (Table 1) and only three patients with metastatic cervical disease. Adverse histopathological risk factors identified at baseline were overall uncommon: positive margin (PM) (n = 1, 5.8%), perineural invasion (PNI) (n = 2, 11.7%), and lymphovascular invasion (LVI) (n = 2, 11.7%). Almost 3/4 of the patients (74%) had a well-differentiated histology identified from their primary tumor.

**Figure 1:**
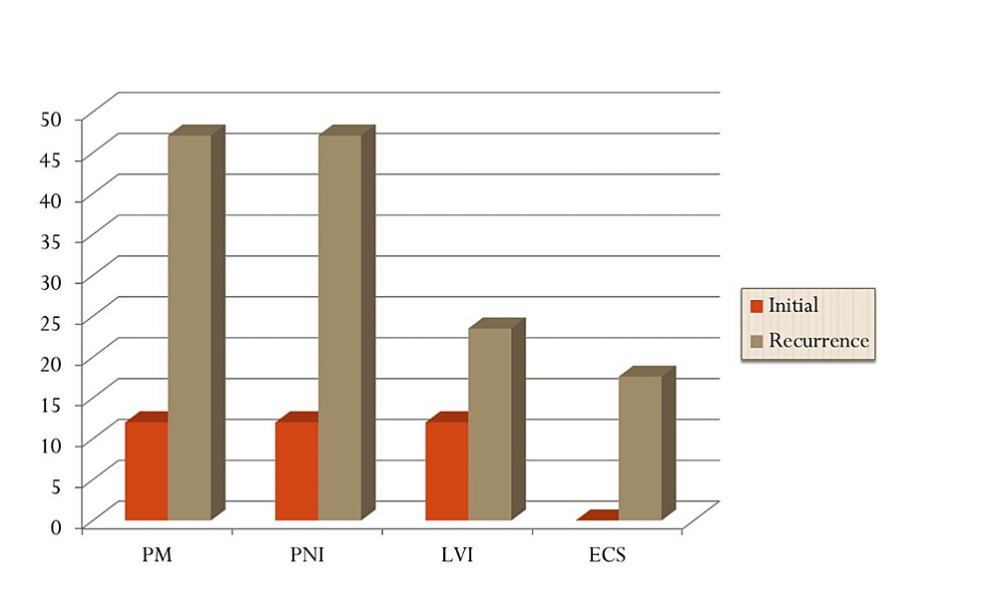
Percentage of patients with high-risk histopathological features by subgroup. ECS: extracapsular spread, PNI: perineural invasion, LVI: lymphovascular invasion, PM: positive margins

Upon recurrence, tumor staging was rT1 = 4 (23.5%), rT2 = 6 (35.3%), rT3 = 2 (11.8%), and rT4 = 5 (29.4%), with seven (41.2%) patients presenting with positive nodal disease, reflective of a higher proportion of advanced stage tumors (outlined in Table 1). Similarly, less than one-third of the patients (29%) had well-differentiated histology, and adverse histopathological features were significantly more common: positive margins (n = 8, 47.1%), PNI (n = 8, 47.1%), LVI (n = 4, 23.5%), and extra-capsular spread (ECS) (n =3, 17.6%). The comparative frequency of adverse histopathological features identified at presentation and upon recurrence is shown in Figure 1.

Oncologic outcomes

Following their initial treatment, the mean locoregional disease-free interval for the entire cohort was 35 months (SD = 19.1 months) and the mean time to reoperation was 35.1 months. After salvage glossectomy, 10 (58.8%) patients failed locoregionally (LR) with a mean LR-disease-free interval of 4.6 months. In this subset of patients, the estimated times for local and nodal recurrence were 15.2 and 22.1 months, respectively. Following their recurrence, the mean follow-up of these patients was 26 months. Kaplan-Meier estimates for one- and three-year disease-free survival (DFS) were 62.7% and 33.4%, while disease-specific survival estimates were 73% and 38.9%, respectively. The mean follow-up for the cohort was 26 months with a range of 2.3-134.2 months (SD = 32.5).

Prognostic factors

**Figure 2:**
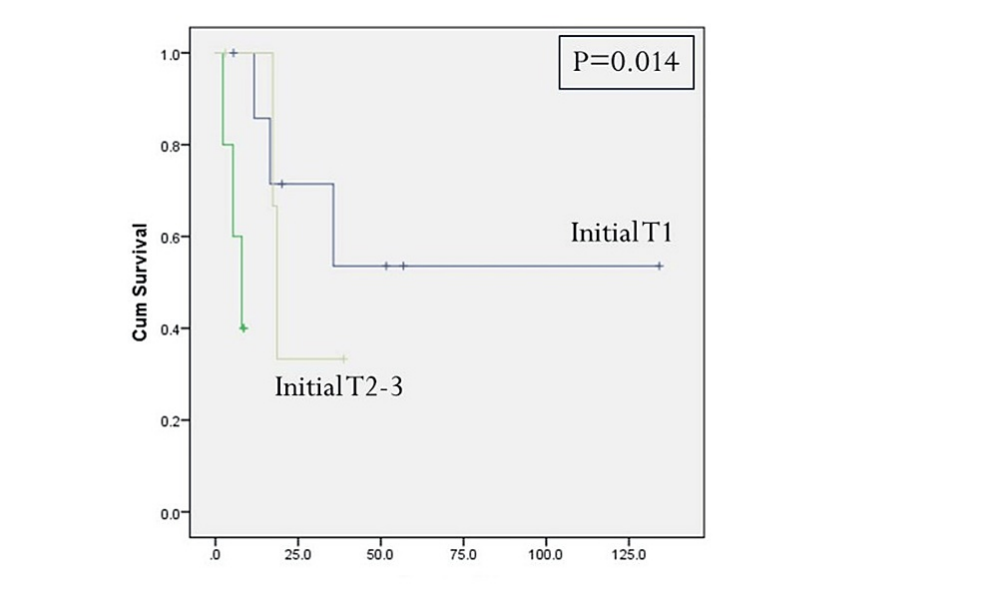
Disease-specific survival based on the initial T-stage. P < 0.05 is considered significant.

**Figure 3:**
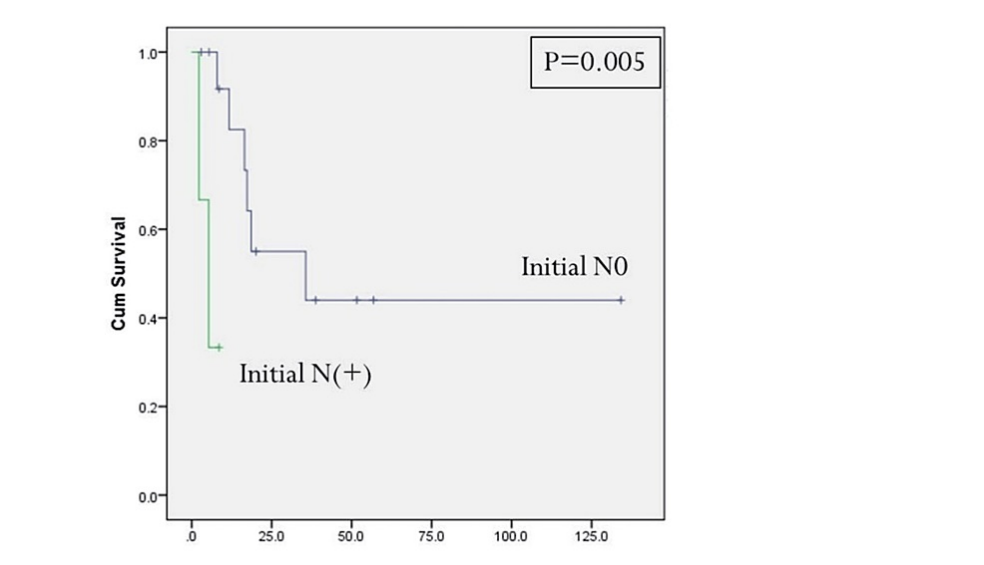
Disease-specific survival based on the initial status. N0: clinically negative neck, N(+): metastatic cervical lymphadenopathy. P < 0.05 is considered significant.

**Figure 4:**
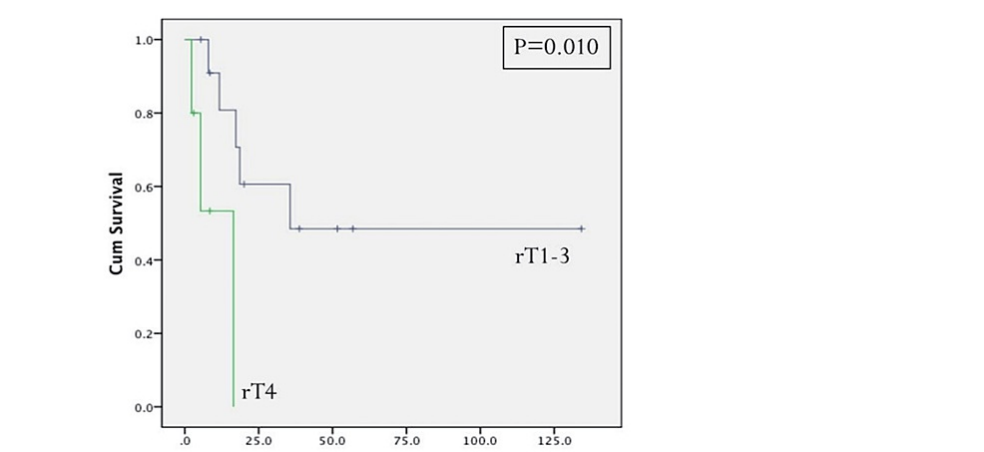
Disease-specific survival based on the recurrent T-status. P < 0.05 is considered significant.

**Figure 5:**
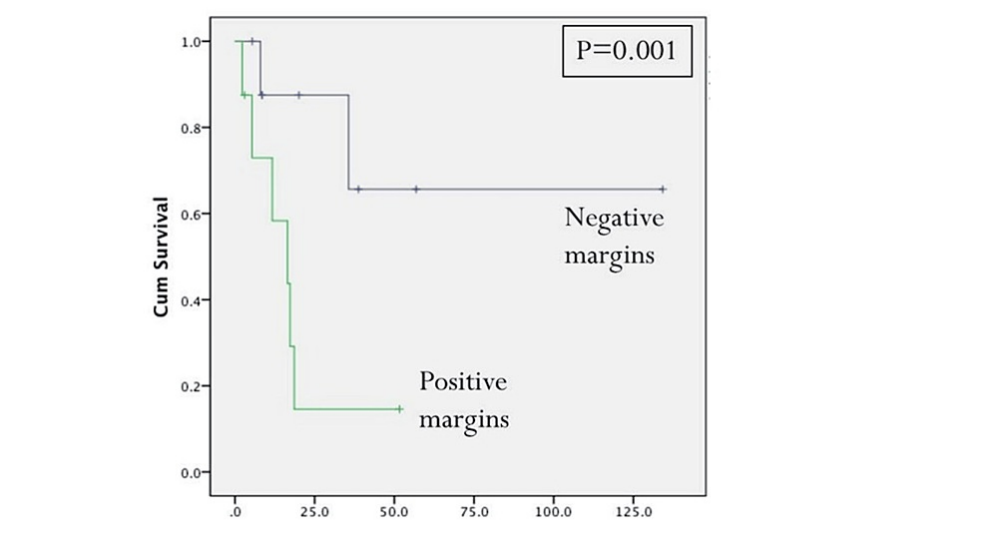
Disease-specific survival based on the recurrent tumor margin status. PM: positive margin. P < 0.05 is considered significant.

**Figure 6:**
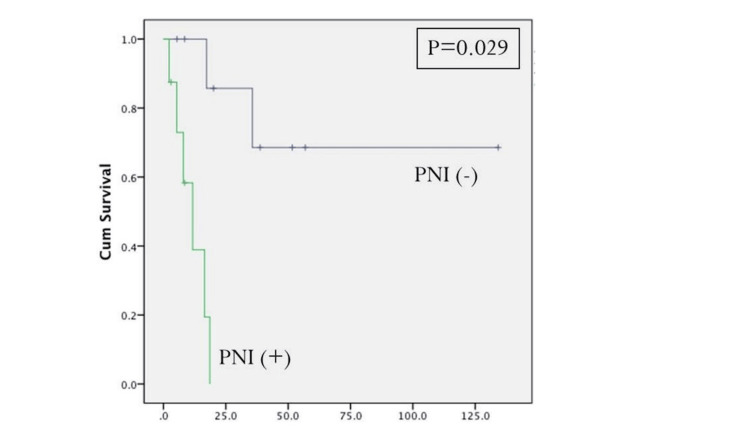
Disease-specific survival based on the presence of recurrent tumor perineural invasion. PNI: perineural invasion. P < 0.05 is considered significant.

Initial tumor T-stage greater T1 and the presence of positive cervical metastatic disease at the time of initial resection were statistically significant predictors for disease-specific survival (Figures 2, 3, Table 4). Positive margins at the time of initial treatment were significantly associated with worse locoregional control (Table 3). At the time of reoperation, recurrent T4-stage (Figure 4), positive margins (Figure 5), and presence of perineural invasion (Figure 6) were all statistically significant predictors for disease-specific survival (Table 4); current alcohol use was associated with worse overall survival (p = 0.024). In contrast to other reports, nodal status at the time of salvage surgery was not a predictor for overall survival. Additional patient- and tumor-related variables tested are described in Tables 3 and 4. 

**Table 3:** Patient demographics and tumor staging at the time of initial presentation and recurrence. N (+): positive cervical metastasis

Demographic	Number	Percentage
Sex		
Male	8	47%
Female	9	53%
Age (mean)	57.8	
Initial tumor stage		
I	8	47%
II	2	12%
III	6	35%
IV	1	6%
Initial N(+) status	3	18%
Recurrent tumor stage		
I	4	24%
II	6	35%
III	2	12%
IV	5	29%
Recurrent N(+) status	7	41%

**Table 4:** Summary of log-rank tests for the initial and recurrent cohort variables' impact on local recurrence. Statistically significant variables are presented in bold. P < 0.05 is considered significant.

Initial cohort variable	P-value	Recurrent cohort variable	P-value
Sex	0.858	Clinical T-stage	0.224
Smoking status	0.627	Glossectomy type	0.429
Alcohol use	0.232	Glossectomy extension	0.429
Clinical T-stage	0.635	Clinical N-stage	0.671
Clinical N-stage	0.938	Clinically positive N-status	0.627
Radiation therapy	0.627	Path T-stage	0.224
Partial glossectomy	1.000	Path N-stage	0.267
Chemoradiotherapy	0.938	Tumor differentiation	0.206
Path T-stage	0.862	Well differentiated	0.901
Path N-stage	0.587	Positive margin	0.232
Positive margins	0.002	Perineural invasion	0.858
Perineural invasion	0.658	Lymphovascular invasion	0.482
Lymphovascular invasion	0.658	Extra-capsular spread	0.159
Advanced stage	0.627	Adjuvant radiotherapy	0.046
Free flap reconstruction	0.858	Adjuvant chemoradiotherapy	0.402

## Discussion

Even in the era of organ preservation therapy, surgical resection with appropriate adjuvant therapy remains the mainstay therapy for primary OTSCC in most institutions. While this approach yields appropriate locoregional control in the majority of patients, those who present with recurrent disease pose a significant challenge for clinicians and are at high risk of succumbing to the disease. When reoperative glossectomy is feasible, salvage surgical attempt is - by all accounts - the preferred treatment modality, commonly in combination with aggressive adjuvant therapy. Despite the oral tongue being the most common subsite for squamous cell carcinoma within the oral cavity, there is a surprising paucity of data regarding prognosis and oncological outcomes in patients undergoing salvage surgery. For clinicians, this translates into limited tools for patient selection, risk stratification, and evidence-based patient counseling.

Data indicate that locoregional recurrence serves as a significant indicator of a poor prognosis in OTSCC, with a dismal 3% three-year actuarial survival rate for patients experiencing local recurrence [10-12]. Yuen et al. later showed that patients with recurrent oral tongue tumors had improved prognosis compared to those managed palliatively, supporting the practice of surgical salvage and raising hope that salvage surgery can benefit patients with recurrent OTSCC [6]. To our knowledge, until recently though, little was known regarding patient- or tumor-specific characteristics that predict oncological outcomes.

Lymph node metastasis serves as a crucial prognostic factor for oral and oropharyngeal carcinomas [13]. Cervical lymph nodes are the primary site for metastasis in OCSCC, significantly reducing the survival rate by 50% [14,15]. In prior times, nodal recurrence was shown to be the most common site of recurrence in oral tongue carcinoma with a greater than 50% nodal recurrence rate for even early-stage tumors [16]. This led to a later work showing that the depth of invasion for oral tongue tumors can be predictive of the rate of occult cervical metastatic disease [17] and subsequently to a more proactive management of the clinically N0 neck, making local recurrence the more relevant site of failure in the modern era.

As in other studies [6], we found that metastatic cervical lymphadenopathy was shown to be significantly associated with worse DSS on initial presentation, but interestingly, this association was not observed in the recurrent setting, despite the fact that 41% of recurrent patients presented with N(+) disease. Conversely, in this subset of patients, we found that advanced T-stage, positive margins, and perineural invasion had an adverse impact on DSS. These results suggest that primary tumor characteristics have a greater impact on survival than metastatic cervical disease at the time of recurrence, which is consistent with the fact that most patients ultimately succumb to an uncontrolled local disease. It is important to note that our study design did capture patients with isolated nodal recurrence, so the true impact of regional failure might be underrepresented in this setting.

The importance of primary tumor characteristics on oncological outcomes appears significant. When considering local recurrence, only the margins from the initial surgery in our population were associated with poorer outcomes. Similarly, the importance of the primary tumor was shown in that the clinical T-stage from the initial cohort and advanced T-stage, positive margins, and perineural invasion at the time of recurrence all resulted in significantly worse disease-specific survival. While extra-capsular spread has been previously shown to be associated with poor oncological outcomes, this was not seen in our study. We did show a statistically significant increase in adverse histopathological findings from the time of the initial tumor to salvage resection, suggestive of dedifferentiation. With many of these features being associated with worse disease-specific survival including positive margins and perineural invasion, it appears that for patients with local recurrence, changes in the tumor biology are the ultimate governors of definitive oncological outcomes.

To our knowledge, our study is the first to show a significant change in the adverse histopathological characteristics from initial presentation to recurrence with a significant impact on local control and survival. These data suggest the importance of recognizing poor histopathological features of the primary and recurrent tumor and matching them with aggressive resection to obtain improved oncological outcomes.

Staging is a critical phase of risk stratification and patient selection, yet for patients with recurrent disease, the prognostic correlation is not as clear as in primary disease. The available evidence is inconsistent in terms of the prognostic implications of both the primary and recurrent staging for patients presenting with recurrence. In a study by Goto et al. [6], survival was related to the stage of the recurrent tumor but not that of the initial tumor, yet other series [18] have shown the opposite, with the primary - but not recurrent - tumor staging being predictors for overall survival. By contrast, in the present series, we found that both initial and recurrent T-stages were statistically significant predictors of survival. While the impact of recurrent staging on oncologic outcomes is somewhat intuitive, explaining the association with the initial tumor staging is harder. We hypothesize that this association might be related to an increased risk of adverse pathological features seen in larger lesions, as well as the increased risk for multifocal or deep tumor recurrences that are seen in larger primary tumors.

Organ preservation therapy is becoming increasingly common in the modern era for the primary management of tumors of multiple head and neck subsites with equal oncological outcomes to surgical resection [19]. Concurrent chemoradiotherapy as an initial treatment for OTSCC has been shown to produce five-year disease survival, ranging from 84.8% to 91.2% [20,21]. Brachytherapy has been shown to result in excellent local tumor control. Early data showed poor oncological outcomes with only a 50% local control rate when performing glossectomy after initial irradiation [22,23]. Mulholland et al. later showed that patients who received initial organ preservation therapy who lateral recurred had similar five-year survival when recurrences were managed with aggressive surgical resection [24]. Similarly, in our study, neither initial organ preservation therapy nor adjuvant radiotherapy was associated with worse locoregional control or disease-specific survival, suggesting that organ preservation approaches can be utilized for OTSCC without negatively impacting long-term survival, even when recurrences do occur. This aligns with the broader understanding that preserving organ function is crucial in head and neck cancer management, not only for anatomical integrity but also for maintaining essential physiological functions and quality of life. 

Recent advancements in surgical techniques, imaging, and adjuvant therapies offer promising prospects for improving salvage surgery outcomes in the future. Integrating innovations, such as minimally invasive approaches, advanced imaging for precise tumor assessment, and tailored adjuvant therapies, could enhance locoregional control and survival rates for patients with recurrent OTSCC. These developments highlight the potential for ongoing research and technological advancements to refine treatment strategies and ultimately improve patient outcomes.

While this study offers valuable insights, it is essential to acknowledge its limitations to provide a comprehensive understanding of the research context. First, the study's single-center design may limit the generalizability of the findings, as results may be influenced by institution-specific practices or patient populations. In addition, the absence of longitudinal patient outcome data restricts our ability to assess the long-term efficacy of salvage surgery and the durability of prognostic factors identified. In other series investigating salvage surgery for oral and oropharyngeal tumors, the time to further locoregional recurrence after the savage surgery has been reported to range from seven to nine months [6,25-27]. In our series, the time to further locoregional recurrence was much shorter at 4.6 months, suggesting that our particular cohort may have presented with a more aggressive disease course or potentially could be the result of more proactive surveillance. Furthermore, inherent biases in retrospective analyses, such as selection bias or incomplete data capture, could impact the reliability and interpretation of the results. For instance, the retrospective nature might influence data accuracy or completeness, potentially leading to underestimation or overestimation of certain prognostic factors. Unfortunately, we had an insufficient number of patients in our study cohort to determine if the increasing incidence of OTSCC in women was reflected in a difference in rates of recurrence. Future studies should attempt to obtain a higher level of evidence through randomizing treatment courses at the time of initial and recurrent OTSCC tumors. As more retrospective series, such as this one, become available, a future meta-analysis will hopefully provide a higher level of evidence to inform clinical decisions.

## Conclusions

Patients with recurrent OTSCC represent a small proportion of patients, but they are at a high risk of succumbing to the disease. Upon recurrence, histopathological analysis reveals detrimental changes in the tumor biology that directly translate to poor disease control. Factors from the initial resection, namely, advanced T-stage, metastatic cervical disease, and positive margins, have a significant impact on local control and survival following salvage surgery. This emphasizes the importance of appropriate treatment upon presentation. The need for aggressive salvage surgery may have influences on preoperative counseling and patient selection for salvage surgery depending on potential functional impairments and signs of recurrence. At the time of salvage surgery, T4 disease, perineural invasion, and positive margin status govern the oncologic outcomes of salvage surgery. Survival rates for patients presenting with any of these findings are dismal despite receiving maximum therapy. Our conclusions are drawn from a retrospective analysis, which, while insightful, necessitates future prospective validation to establish causality more definitively. Furthermore, our findings suggest that aggressive salvage surgery is critical for improving outcomes in recurrent OTSCC. However, further multi-center, prospective studies are needed to generalize these conclusions across diverse patient populations and care settings.
